# A comparison of the effects of NOAC and VKA therapy on the incidence of dementia in patients with atrial fibrillation: A systematic review and meta‐analysis

**DOI:** 10.1002/clc.24076

**Published:** 2023-06-27

**Authors:** Wenjie Wang, Weiguo Fan, Yuhao Su, Kui Hong

**Affiliations:** ^1^ Department of Cardiovascular Medicine The Second Affiliated Hospital of Nanchang University Nanchang Jiangxi China; ^2^ Jiangxi Key Laboratory of Molecular Medicine Nanchang Jiangxi China; ^3^ Department of Genetic Medicine The Second Affiliated Hospital of Nanchang University Nanchang Jiangxi China

**Keywords:** anticoagulation, atrial fibrillation, dementia, meta‐analysis, nonvitamin K antagonist oral anticoagulant, vitamin K antagonist

## Abstract

Atrial fibrillation (AF) patients are more susceptible to dementia, but the results about the effect of oral anticoagulants (OACs) on the risk of dementia are not consistent. We hypothesize that OAC is associated with a reduced risk of dementia with AF and that nonvitamin K antagonist oral anticoagulants (NOAC) are superior to vitamin K antagonists (VKA). Four databases were systematically searched until July 1, 2022. Two reviewers independently selected literature, evaluated quality, and extracted data. Data were examined using pooled hazard ratios (HRs) and 95% confidence intervals (CIs). Fourteen research studies involving 910 patients were enrolled. The findings indicated that OACs were associated with a decreased risk of dementia (pooled HR: 0.68, 95% CI: 0.55–0.82, *I* ^2^ = 87.7%), and NOACs had a stronger effect than VKAs (pooled HR: 0.87, 95% CI: 0.79–0.95, *I* ^2^ = 72%), especially in participants with a CHA2DS2VASc score ≥ 2 (pooled HR: 0.85, 95% CI: 0.72–0.99). Subgroup analysis demonstrated no statistical significance among patients aged <65 years old (pooled HR: 0.83, 95% CI: 0.64–1.07), patients in “based on treatment” studies (pooled HR: 0.89, 95% CI: 0.75–1.06), or people with no stroke background (pooled HR: 0.90, 95% CI: 0.71–1.15). This analysis revealed that OACs were related to the reduction of dementia incidence in AF individuals, and NOACs were better than VKAs, remarkably in people with a CHA2DS2VASc score ≥ 2. The results should be confirmed by further prospective studies, particularly in patients in “based on treatment” studies aged <65 years old with a CHA2DS2VASc score < 2 or without a stroke background.

## Graphical abstract

Atrial fibrillation patients are more susceptible to dementia. Oral anticoagulants (OAC) were associated with a decreased risk of dementia, and nonvitamin K antagonist oral anticoagulants (NOACs) had a stronger effect than vitamin K antagonists (VKAs). A summary of point estimates of hazard ratios, 95% confidence intervals, and *p* values were were shown, and a random effects model was used.

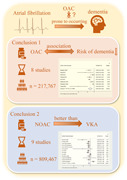

## 1 INTRODUCTION

Atrial fibrillation (AF) is an increasingly prevalent arrhythmia that represents a substantial load on citizens and healthcare systems across the world; additionally, it causes various complications, including heart failure and vascular thromboembolic events.^1^, ^2^, ^3^ Dementia can result in cognitive impairment rather than a specific illness; the predominant forms of dementia consist of Alzheimer's disease (AD) and vascular dementia.^4^ Microembolism developed in AF patients may lead to intracranial circulation disturbance, while decreased cardiac output may worsen cerebral hypoperfusion. Additionally, anticoagulation therapy may heighten the risk of hemorrhage and cause microbleeds inside the brain. All of these factors, with the addition of stroke, are strongly associated with cognitive disorders.^5^, ^6^

Increasing evidence has shown that the dementia risk is 1.4 times greater in AF patients than in non‐AF patients, 2.7 times greater in AF patients with stroke,^7^ and 27% greater in AF patients without stroke.^8^ Accordingly, additional investigations are necessary to examine whether appropriate management of AF might have the potential to impede or postpone the emergence of dementia. Some guidelines recommend the use of oral anticoagulants (OACs) to prevent stroke among AF patients.^9^ Additionally, there is evidence that OACs are linked to a decreased prevalence of dementia, thus indicating that early anticoagulation therapy might be beneficial for cognitive protection.^10^, ^11^, ^12^, ^13^

Compared with traditional OACs, such as warfarin, a kind of vitamin K antagonist (VKA), the nonvitamin K antagonist oral anticoagulant (NOAC) has been shown to have the advantages of a stable anticoagulant effect, rapid onset of action, fewer individual differences, and a lower risk of intracranial hemorrhage complications. A more current study demonstrated that NOAC was more effective than VKA in reducing the overall incidence of dementia.^14^ However, it should be noted that the results of this study were derived from numerous randomized controlled trials (RCTs), in which dementia was reported as an adverse effect and the definition was unclear. Furthermore, several large cohort studies did not reveal a notable disparity between NOACs and VKAs.^13^, ^15^, ^16^, ^17^ Thus, it remains unclear whether NOAC or VKA is superior for preventing dementia in AF patients.

To date, doctors may choose to temporarily offer OACs to those with normal cognition, which might increase the number of dementia diagnoses soon after the patient's registration. Therefore, dementia diagnosed at the beginning of follow‐up, known as the “blank period,” should be excluded. Unfortunately, the pooled risk ratio of studies with an observational blank period was not statistically significant.^18^ Moreover, varying definitions of anticoagulation were used when grouping in a number of cohort studies, which may directly affect the statistical results of the study. Regretfully, there was no comprehensive study being conducted to examine this matter.

Therefore, in light of the seven most recent studies, a systematic review and meta‐analysis were carried out to explore the beneficial effects of anticoagulant medication for predicting dementia in AF patients and to determine whether NOACs are more effective than VKAs. In addition, the effects were evaluated via subgroup analyses based on age, CHA_2_DS_2_‐VASc score, the inclusion or exclusion of a blank period, the inclusion or exclusion of stroke patients, and the definition of anticoagulation.

## 2 METHODS

The Preferred Reporting Items for Systematic Reviews and Mete‐Analyses (PRISMA) standards were followed for conducting this study,^19^ as shown in Supporting Information: Table 1. It was not registered in PROSPERO.

### 2.1 Data sources and search strategy

To find all qualifying studies from their beginning to July 1, 2022, the online databases of PubMed, Embase, Cochrane Library, and Web of Science were retrieved with no language restrictions. Four components made up the bulk of the search technique for terms: AF; anticoagulant or indirect thrombin inhibitor; dementia or AD or vascular dementia or cognition disorder; drug therapy or RCT or controlled clinical trial or cohort study; their Medical Subject Heading entry words were checked as well. To augment the theoretically relevant studies, reference lists from each enrolled article and prior meta‐analysis were painstakingly retrieved. The literature search terms and strategies are displayed in Supporting Information: Table 2.

### 2.2 Selection criteria

Two authors (W. W. and W. F.) separately searched for articles using a standard procedure. In the event of discordance between them, another researcher (Y. S.) provided suggestions until a consensus was reached. The retrieved articles were arranged using Endnote Version x9.1.

The inclusion criteria were as follows: (a) AF patients older than 18 years; (b) probing the influence of VKAs or NOACs on dementia risk; (c) presenting the results using a hazard ratio (HR) and 95% confidence intervals (CIs); (d) RCTs or cohort studies; and (e) dementia or other relevant cognitive declines as the primary endpoints.

The following were the exclusion requirements: (a) valvular AF; (b) individuals with prior events of moderate to severe cognitive impairment or dementia at baseline; (c) only rough or unadjusted HR; (d) cross‐sectional studies; and (e) articles with following types: conference abstract, editorial material, letter, expert opinion, case report, or review article.

OACs encompassed VKAs and NOACs; VKA referred to warfarin and NOACs included dabigatran, rivaroxaban, apixaban, and so forth; non‐OAC encompassed antiplatelet medications or absence of antithrombotic treatment; dementia encompassed AD, vascular dementia, and other types of cognition disorders. The study with the most comprehensive and up‐to‐date data was selected for overlapping participants. The primary outcomes of the study were the overall occurrences of dementia.

### 2.3 Data extraction and quality assessment

The data extracted from the enrolled articles were separately carried out by two authors (W. W. and W. F.), which consisted of the primary author's name, year of publication, geographical location, data sources, the year of the data collected, number of patients, types of drugs, comparison group, patients' average age, percentage of females, CHA_2_DS_2_‐VASc score, follow‐up time, diagnosis for dementia, whether the stroke was excluded, blank period, definition of anticoagulant treatment, adjusted or matching variables, and HR with 95% CIs. The differences were resolved through negotiation by two authors and subsequently verified by the third author (Y. S.) upon review.

The quality of the cohort studies included was assessed with the Newcastle–Ottawa Quality Assessment Scale (NOS).^20^ Three subscales totaling nine items, each earning one point, made up the NOS. Low, moderate, and excellent quality are represented by scores ranging from 0 to 3, 4 to 6, and 7 to 9, respectively. Two authors (W. W. and W. F.) separately completed quality assessments, with discrepancies resolved by another author (Y. S.).

### 2.4 Statistical analysis

The HR and 95% CIs for each risk factor were aggregated and evaluated using Stata 17.0. Heterogeneity was taken into account while choosing the pooled data for the model and was evaluated using *I* ^2^ statistics and *p* values. A random‐effects model was used to examine data with high heterogeneity (classified as *I* ^2^ > 50% or *p* < .1), whereas a fixed‐effects model was employed in other situations.^21^ To investigate the possible effect of heterogeneity, subgroup analyses were conducted based on age stratification (<75 vs. ≥75 years and <65 vs. ≥65 years), CHA_2_DS_2_‐VASc score (<2 vs. ≥2), presence of a blank period, exclusion of stroke, the definition of anticoagulation (studies in which patients received anticoagulants for more than a certain proportion of time during the follow‐up period were classified as “based on treatment,” while studies in which patients were classified according to their initial prescription were classified as “intention to treat”), and specific types of NOAC. Publication biases were assessed with funnel plots, examining with Begg's and Egger's tests.

## 3 RESULTS

### 3.1 Study selection

**Figure 1:**
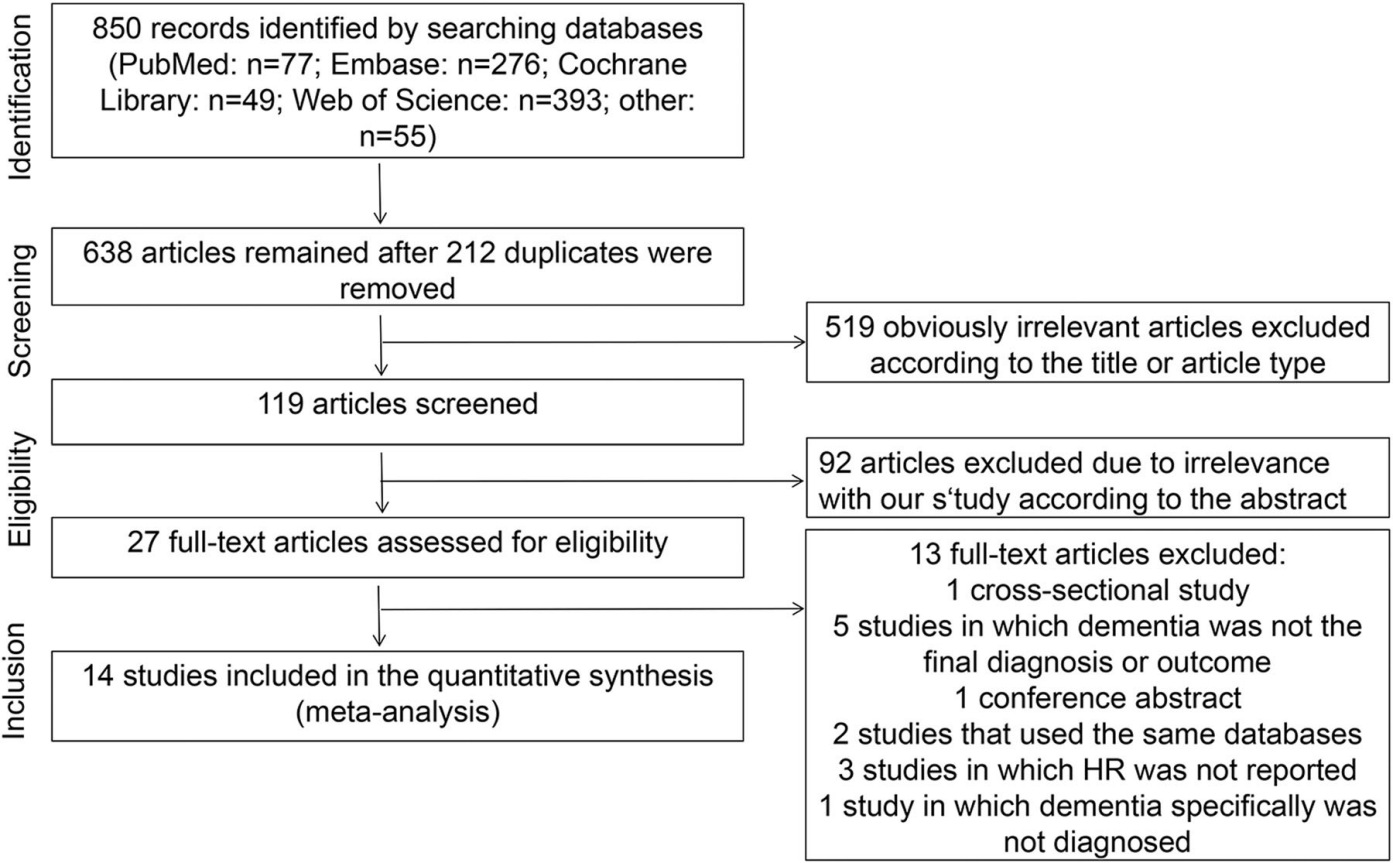
PRISMA flowchart. PRISMA, Preferred Reporting Items for Systematic Reviews and Meta‐analyses; HR, hazard ratio.

Above all 850 entries were found in the online database. Following deleting 212 duplicate records, reading the headings and abstracts, and applying screening criteria, 27 articles were found to be eligible. Therein, some relevant RCTs were excluded because dementia was reported as an adverse side effect rather than as the primary outcome, as shown in Supporting Information: Table 3.^22^, ^23^, ^24^, ^25^ Ultimately, 14 articles were enrolled into our meta‐analysis.^8^, ^10^, ^11^, ^12^, ^13^, ^15^, ^16^, ^17^, ^26^, ^27^, ^28^, ^29^, ^30^, ^31^ The PRISMA flowchart of this study filtering process is displayed in Figure 1.

All 14 included articles were retrospective studies, including 5 studies from Asia,^8^, ^15^, ^28^, ^29^, ^31^ 6 from Europe,^10^, ^11^, ^13^, ^17^, ^27^, ^30^ 2 from North America,^12^, ^16^ and 1 from Oceania,^26^ with a total of 910 200 patients. The basic information for each study is shown in Table 1. All articles were deemed as being of good quality based on the NOS scale, as detailed in Supporting Information: Table 4.

**Table 1:** Basic characteristics of all enrolled studies.

Study (first author, year)	Data source	Duration of data collection	Groups	Sample	Mean Age, years	Female, %	CHA_2_DS_2_‐VASc	Follow‐up duration, years	Incidence, /1000 person‐year	Diagnostic for dementia (all cause)	Excluded stroke	Excluded dementia	Banking period	Definition of anticoagulation	Adjusted or matching variables
Bezabhe 2022	Australia‐wide primary care data	2010–2017	OAC versus non‐OAC	18 813	71.9	47.1	2.9	3.7	6.1	Comorbid code terms of Medicine Insight Data Dictionary	Yes	Yes	1 year	Based on treatment	Sex, age, duration of follow‐up, comorbidities and medications
			NOAC versus VKA	8243	73.9	45.9	3.0	3.7	5.5						
Cadogan 2021	UK Clinical Practice Research Datalink, Hospital Episodes Statistics and Index of Multiple Deprivation	2012–2018	NOAC versus VKA	39 200	76	44.6	NA	NA	VKA: 12.5	Read codes	No	Yes	1 year	Intention to treat	Age, calendar year, time‐on‐treatment, sex, BMI, smoking status, hazardous alcohol consumption, socioeconomic status (practice level Index of Multiple Deprivation), primary care consultation frequency, comorbidities and medications
									NOAC: 11.0						
Chen 2018	US healthcare claim databases, MarketScan (2007–2015), Optum Clinformatics (2009–2015)	2007–2015	NOAC versus VKA	468 445	70	35	3	0.7–2.2	VKA: 10.5	ICD‐9	No	Yes	No	Intention to treat	Age, sex, race, education level, household income level, and prevalent cognitive impairment, comorbidities and medications
									NOAC: 8.7						
Ding 2018	Swedish National Study on Aging and Care in Kungsholmenn	2001–2013	OAC versus Non‐OAC	470	≥60	NA	NA	6	NA	DSM‐IV criteria	No	Yes	No	Not reported	Age, sex, education, ever smoking, excessive alcohol consumption, physical inactivity, BMI, and comorbidities
Field 2019	UK Clinical Practice Research Datalink	2008–2016	OAC versus non‐OAC	6190	NA	NA	NA	2	OAC: 13.7	Read codes and ICD‐10	No	Yes	No	Based on treatment	NA
									Non‐OAC: 14.0						
Friberg 2019	Swedish Patient register	2006–2014	OAC versus non‐OAC and NOAC versus VKA	91 254	60	30	0.9	4.7	OAC: 1.6	ICD‐10	Yes	Yes	1 month	Both	Age, sex, CHA_2_DS_2_‐VASc, Alcohol index, frequent falls, comorbidities and medications
									Non‐OAC: 2.7						
Hsu 2021	The Taiwan National Health Insurance Research Database	2012–2016	NOAC versus VKA	25 089	70.3	40.5	2.9	3.27	VKA: 19.4	ICD‐9 and ICD‐10	No	Yes	3 months	Based on treatment	Age, sex, CHA_2_DS_2_‐VASc, HAS‐BLED, income level, index year, interval between AF diagnosis and OAC use, comorbidities and medications
									NOAC: 15.4						
Kim 2019	Korea National Health Insurance Service Senior cohort	2005–2012	OAC versus non‐OAC	10 435	71.7	47.3	2.3	7.2	OAC: 2.1	ICD‐10	Yes	Yes	No	Not reported	Age, sex, CHA_2_DS_2_‐VASc, BMI, blood pressure, blood glucose, total cholesterol, serum creatinine, economic status, smoking status, alcohol consumption, comorbidities and medications
									Non‐OAC: 5.0						
Kim 2020	Korea National Health Insurance Service Senior cohort	2013–2016	NOAC versus VKA	53 236	70.7	41.3	4	20.2	VKA: 5.0	ICD‐10	No	Yes	6 months	Intention to treat	Age, sex, CHA_2_DS_2_‐VASc, HAS‐BLED, income, frailty, AF duration, comorbidities and medications
									NOAC: 3.5						
Lee 2021	Korean nationwide claims database	2014–2017	NOAC versus VKA	72 846	71.8	42	4	1.3	VKA: 4.7	ICD‐10	No	Yes	No	Intention to treat	Age, sex, CHA_2_DS_2_‐VASc, smoking status and comorbidities
									NOAC: 5.0						
Madhavan 2018	Rochester Epidemiology Project	2000–2010	OAC versus non‐OAC	2800	71.2	46.6	3	5	NA	ICD‐9	No	Yes	6 months	Intention to treat	Age, sex, BMI, history of smoking, and comorbidities
Mongkhon et 2020	UK primary care data	2000–2017	OAC versus non‐OAC	84 521	NA	NA	NA	5.9	OAC: 12.1	Read codes or antidementia drugs	No	Yes	1 year	Intention to treat	NA
									Non‐OAC: 13.3						
			NOAC versus VKA	17 537	NA	NA	NA	3.4	VKA: 12.9						
									NOAC: 12.8						
Søgaard 2019	Danish National Patient Register Danish National Prescription Register Danish Civil Person Register	2011–2016	NOAC versus VKA	33 617	>60	NA	NA	3.4	NA	ICD‐8 and ICD‐10	Yes	Yes	6 months	Based on treatment	Age, sex, CHA_2_DS_2_‐VASc, HAS‐BLED, comorbidities and medications
Wong 2020	AF registry of Queen Mary Hospital, Hong Kong	1997–2011	OAC versus non‐OAC	3284	76.4	48.4	3.94	3.6	NA	Disease codes	Yes	Yes	2 weeks	Intention to treat	Age, sex, CHA_2_DS_2_‐VASc and comorbidities

Abbreviations: AF, atrial fibrillation; BMI, body mass index; DSM, the diagnostic and statistical manual of mental disorders; ICD, International Classification of Diseases; NA, not available; NOAC, nonvitamin K antagonist oral anticoagulant; OAC, oral anticoagulant; UK, United Kingdom; US, United States; VKA, vitamin K antagonist.

### 3.2 The effect of OACs on the risk of dementia in AF patients

**Figure 2:**
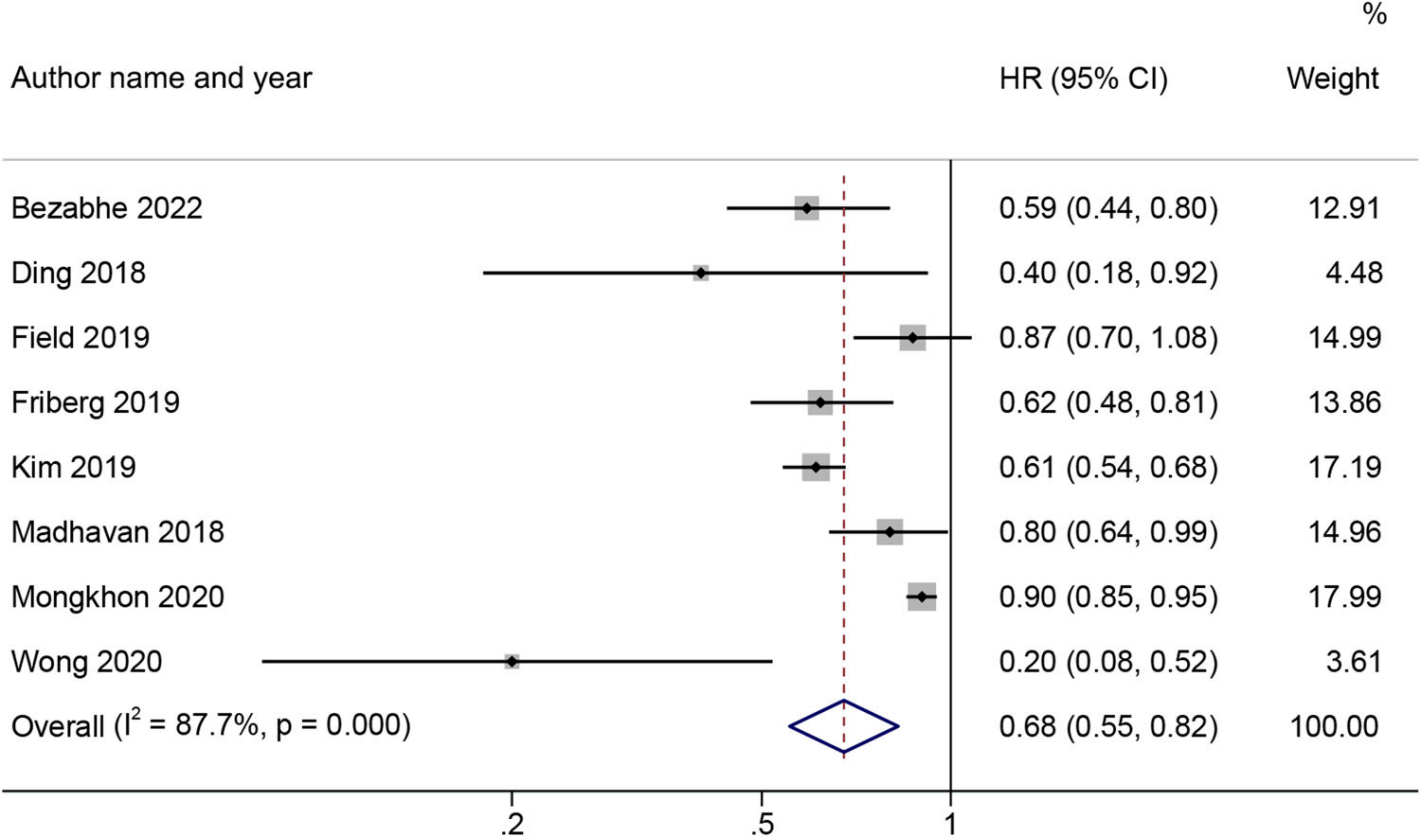
Risk of dementia in AF patients treated with OACs and non‐OACs. AF, atrial fibrillation; CI, confidence interval; HR, hazard ratio; OAC, oral anticoagulant.

As shown in Figure 2, the dementia risk in AF patients was compared between patients with OACs and non‐OACs in 8 studies^8^, ^10^, ^11^, ^12^, ^13^, ^26^, ^29^, ^30^ (*n* = 217 767). The results suggested that the application of OACs was associated with a decreased dementia risk in comparison to non‐OACs (pooled HR: 0.68, 95% CI: 0.55–0.82), which adopted a random‐effects model to combine data because of the high level of heterogeneity (*I* ^2^ = 87.7%, *p* < .001).

### 3.3 The effect of NOAC on the risk of dementia in AF patients compared with VKA

**Figure 3:**
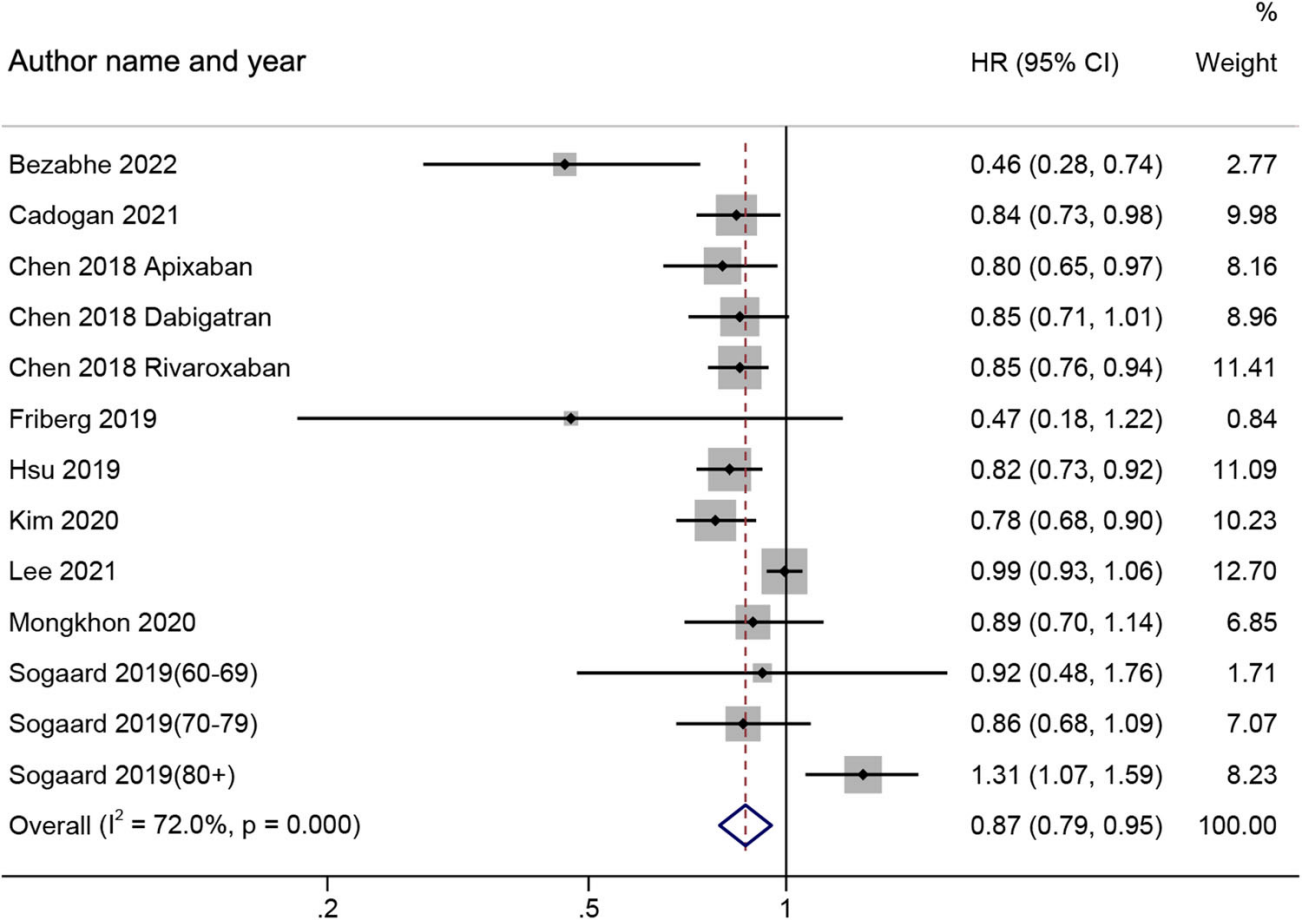
Risk of dementia in AF patients treated with NOAC and VKA. AF, atrial fibrillation; CI, confidence interval; HR, hazard ratio; NOAC, nonvitamin K antagonist oral anticoagulant; VKA, vitamin K antagonist.

As shown in Figure 3, the prevalence of dementia in AF individuals was compared between NOAC and VKA in nine studies^11^, ^13^, ^15^, ^16^, ^17^, ^26^, ^27^, ^28^, ^31^ (*n* = 809 467). The pooled analysis indicated that people receiving NOAC treatment were related to a potential reduction of dementia risk than those treated with VKA (pooled HR: 0.87, 95% CI: 0.79–0.95). A random‐effects model was employed since there is a considerable variability among research (*I* ^2^ = 72%, *p* < .001).

### 3.4 Subgroup analyses

Several subgroup analyses were performed because of the substantial level of heterogeneity when comparing OACs with non‐OACs and NOACs with VKAs (Table 2).

**Table 2:** Subgroup analyses: OACs and incidence of dementia in AF patients.

Groups	Subgroups	Number of studies	Sample	Pooled HR (95% CI)	Model	*I* ^2^	*p*
OAC versus non‐OAC	CHA_2_DS_2_‐VASc						
	<2	3	63 962	0.77 (0.57–1.04)	Random	70.0%	.036
	≥2	3	66 172	0.94 (0.89–0.99)	Fixed	0.0%	.383
	Blank period						
	Yes	5	136 408	0.68 (0.52–0.87)	Random	83.6%	<.001
	No	3	43 212	0.67 (0.49–0.93)	Random	78.8%	.009
	Stroke was excluded						
	Yes	4	59 533	0.60 (0.54–0.66)	Fixed	44.9%	.142
	No	4	120 087	0.89 (0.84–0.94)	Fixed	37.6%	.187
	Definition of anticoagulation						
	Based on treatment	3	78 099	0.71 (0.62–0.83)	Random	66.0%	.053
	Intention to treat	4	138 097	0.79 (0.65–0.96)	Random	75.8%	.006
	CHA_2_DS_2_‐VASc						
NOAC versus VKA	<2	5	48 459	0.70 (0.46–1.06)	Fixed	0.0%	.667
	≥2	5	150 389	0.85 (0.72–0.99)	Fixed	34.3	.193
	Age						
	<75	7	247 331	0.78 (0.72–0.85)	Fixed	0.0%	.892
	≥75	6	131 260	0.89 (0.82–0.96)	Random	57.4%	.016
	<65	2	20 707	0.83 (0.64–1.07)	Fixed	41.2%	.192
	≥65	3	65 553	0.79 (0.64–0.98)	Random	84.9%	<.001
	Blank period						
	Yes	9	131 600	0.84 (0.73–0.98)	Random	71.6%	<.001
	No	5	390 472	0.88 (0.80–0.97)	Random	66.7%	.017
	Stroke was excluded						
	Yes	6	96 534	0.90 (0.71–1.15)	Random	76.6%	.001
	No	8	440 463	0.86 (0.79–0.93)	Random	64.4%	.006
	Definition of anticoagulation						
	Based on treatment	7	127 323	0.89 (0.75–1.06)	Random	79.7%	<.001
	Intention to treat	7	402 447	0.90 (0.84–0.96)	Random	74.5%	<.001
	NOAC						
	Apixaban	3	79 118	0.85 (0.66–1.10)	Random	85.9%	.001
	Dabigatran	3	109 219	0.90 (0.84–0.97)	Fixed	30.0%	.240
	Rivaroxaban	3	148 118	0.85 (0.76–0.96)	Random	67.6%	.046

Abbreviations: AF, atrial fibrillation; CI, confidence interval; HR, hazard ratio; NOAC, nonvitamin K antagonist oral anticoagulant; OAC, oral anticoagulant; VKA, vitamin K antagonist.

In the subgroup analyses, the correlation of OACs and the dementia risk was unclear among patients with CHA_2_DS_2_‐VASc < 2 (pooled HR: 0.77, 95% CI: 0.57–1.04) but displayed a downward trend among patients with CHA_2_DS_2_‐VASc ≥ 2 (pooled HR: 0.94, 95% CI: 0.89–0.99), and there was mild heterogeneity. Additionally, regardless of whether there was a blank period, whether a previous stroke population was enrolled, or whether a different definition of anticoagulation was used, the protective effects of OACs remained consistent.

In the comparison between the use of NOAC and VKA to prevent dementia, the results suggested a reduction in patients with CHA_2_DS_2_‐VASc ≥ 2 (pooled HR: 0.85, 95% CI: 0.72–0.99). Nevertheless, there was no statistically significant variation in patients aged <65 (pooled HR: 0.83, 95% CI: 0.64–1.07), when stroke patients were excluded (pooled HR: 0.90, 95% CI: 0.71–1.15) or when patients were grouped based on treatment (pooled HR: 0.89, 95% CI: 0.75–1.06). Furthermore, when comparing different types of NOACs with VKA, dabigatran and rivaroxaban usage was markedly associated with a decline in dementia incidence, while the use of apixaban manifested no similar effects.

### 3.5 Publication bias

In the comparison of OACs with non‐OACs, no obvious publication bias was observed according to the results of Begg's test (*p* = .174) and Egger's test (*p* = .055). Similarly, when comparing NOACs with VKA, no obvious publication bias was found based on Begg's test (*p* = .951) and Egger's test (*p* = .129). The funnel plots are shown in Supporting Information: Figures 1 and 2.

## 4 DISCUSSION

### 4.1 Summary of evidence

The aim of our research was to investigate the effect of OACs on dementia risk in AF patients and to determine whether NOACs are more effective than VKAs. In addition, 14 large retrospective cohort studies with representative sample sizes were included in this review. One novel aspect of this study was that we not only provided an update on the seven most recent studies^8^, ^15^, ^26^, ^27^, ^28^, ^29^, ^31^ but also conducted subgroup analyses based on factors such as age, CHA_2_DS_2_‐VASc score, presence of a blank period, exclusion of stroke patients, the definition of anticoagulation, and specific type of NOAC. These subgroup analyses help to maximize the validity of the findings, thereby yielding more reliable and up‐to‐date evidence.

Our findings suggested that OACs were associated with a protective effect against dementia in AF persons, and NOACs were superior to VKAs. Other relevant theories have been put up, despite the fact that the precise mechanism that underlies the connection between AF and dementia has not yet been completely elucidated. The impact of subclinical cerebrovascular disease and inadequate cerebral perfusion, especially reduced blood flow to the temporal lobe, is currently the prevailing theory; this mechanism can be prevented by the use of OACs.^32^, ^33^ Additionally, NOACs hinder thrombin function by inhibiting its function (dabigatran) or generation (factorXa inhibitors, e.g., apixaban and rivaroxaban), while thrombin plays a critical role as a dementia‐related intermediary of cognitive and neurological impairment; therefore, administering NOACs may help to maintain vascular integrity and cerebral blood flow, potentially mitigating neurocognitive impairment caused by vascular disease.^34^ Moreover, crucial clinical studies have demonstrated that NOACs, when compared with VKAs, are capable of avoiding ischemic stroke or thromboembolism and are correlated with a decreased incidence of cerebral hemorrhage.^22^, ^24^, ^25^ Nevertheless, the effect among different NOACs may vary. Previous studies comparing NOAC to VKA have shown that dabigatran and apixaban have superior efficacy and safety.^35^, ^36^ However, our results only showed that dabigatran and rivaroxaban had a clear protective effect on the risk of dementia. Therefore, it is necessary to conduct additional investigations to determine whether different NOACs have varying risks of causing silent or subclinical strokes and whether these effects are linked to cognitive function.

In addition, the results showed that a trend toward a reduced dementia danger was more pronounced in individuals with a CHA_2_DS_2_‐VASc score ≥ 2 and age ≥ 65. The CHA_2_DS_2_‐VASc is a tool that is used to evaluate the risk of embolism, and age is one of the scoring items. In the latest clinical guidelines, anticoagulant therapy was recommended for patients with a score ≥ 2, as higher scores indicate a greater risk of embolism,^37^ while NOACs minimize the incidence of intracranial bleeding without raising the danger of ischemia.

Additionally, when stroke participants were taken out of the study, the efficacy of NOAC prophylaxis on dementia in AF individuals was unclear compared with VKA. Strokes have been proven to double the danger of dementia.^38^ Obviously, the result was more credible when examining participants without a stroke background. However, it was revealed that those with AF might have a greater dementia danger, which was independent of strokes or acute ischemic events.^39^ Thus, further investigations are required to corroborate the outcomes.

Furthermore, our subgroup analysis showed that NOAC had a stronger protective effect than VKA regardless of whether a blank period was included, which was different from the findings of a previous meta‐analysis.^18^ Indeed, dementia cases were more likely to appear within the initial year of monitoring, suggesting that the participants were already in the prodromal stage of dementia development before they were enrolled.^40^ Thus, the blank period may help to minimize the overestimation of pharmacological protective effects. In light of this, the pooled HR with a blank period might offer a more accurate representation of the real effect of OACs or NOACs on dementia danger.

Moreover, regardless of the definition of anticoagulation used, the reduction of OACs on dementia risk in AF individuals was confirmed. However, this protective effect did not reach statistical significance when comparing NOAC to VKA in the “based on treatment” group, which guaranteed the duration of action of the drug and was more likely to reflect the actual effect of the drugs in reducing dementia risk in AF patients.

We excluded some RCTs because they reported dementia as an adverse side effect rather than the primary outcome. Additionally, there are several ongoing RCTs related to this topic that may provide further insights and clarify our uncertain conclusions, as listed in Supporting Information: Table 5. We will continue to monitor these studies and update our findings as necessary.

### 4.2 Limitations

Our research still had some limitations that should be acknowledged. First, while we used random effects models, most of the analyses within each group had a high level of heterogeneity, which could be attributed to differences in screening criteria, sample sizes, demographics of the study population, types of drugs studied, follow‐up times, unmeasured confounding factors, and other variables. The factors utilized in every trial for statistical adjustment might have also attributed to the significant level of heterogeneity. Additionally, the present included data were observational studies with several potential biases that we were unable to control or measure. For instance, whether or what kind of medication the patient chooses might be influenced by other factors such as socioeconomics, which will affect the incidence of dementia. Furthermore, due to the limited duration of follow‐up in most studies, it cannot be guaranteed that the incidence of dementia was accurately assessed. Dementia is a slow‐progressing disease, the development of which is influenced by age as a standalone risk factor, which could be unrelated to the diagnosis of AF. This makes establishing a logical link between them challenging. These limitations should be considered when interpreting our findings.

## 5 CONCLUSIONS

The results of this systematic review and meta‐analysis, which comprised 14 retrospective cohort studies, suggested an association between OACs and a lower risk of dementia in AF patients. Additionally, NOAC was more effective than VKA, regardless of whether a blank period was included in the analysis, as well as in persons with a CHA_2_DS_2_‐VASc score ≥ 2. However, the impact could not be determined, particularly in subpopulations such as those in “based on treatment” studies, those aged <65 years old, those with a CHA_2_DS_2_‐VASc score < 2, or those without a stroke history. Therefore, NOACs should be considered a preferred first‐line option in AF individuals to minimize the dementia danger unless other factors outweigh the risk in favor of VKA. Accordingly, more high‐quality RCTs and cohort studies are expected to clarify the dementia risk in AF patients receiving various anticoagulants and to provide better guidance for clinical practice.

## CONFLICT OF INTEREST STATEMENT

The authors declare no conflict of interest.

## Supporting information

Supporting information.

## Data Availability

The data supporting this meta‐analysis are from previously reported studies and datasets, which have been cited.
